# Utility of monocyte HLA-DR and rationale for therapeutic GM-CSF in sepsis immunoparalysis

**DOI:** 10.3389/fimmu.2023.1130214

**Published:** 2023-02-07

**Authors:** Ila Joshi, Walter P. Carney, Edwin P. Rock

**Affiliations:** ^1^ Development and Regulatory Department, Partner Therapeutics, Inc., Lexington, MA, United States; ^2^ Walt Carney Biomarkers Consulting, LLC., North Andover, MA, United States

**Keywords:** sepsis, immunoparalysis, immunosuppression, granulocyte-macrophage colony-stimulating factor, human leukocyte antigen-DR, monocytes, compensatory anti-inflammatory response syndrome, sargramostim

## Abstract

Sepsis, a heterogeneous clinical syndrome, features a systemic inflammatory response to tissue injury or infection, followed by a state of reduced immune responsiveness. Measurable alterations occur in both the innate and adaptive immune systems. Immunoparalysis, an immunosuppressed state, associates with worsened outcomes, including multiple organ dysfunction syndrome, secondary infections, and increased mortality. Multiple immune markers to identify sepsis immunoparalysis have been proposed, and some might offer clinical utility. Sepsis immunoparalysis is characterized by reduced lymphocyte numbers and downregulation of class II human leukocyte antigens (HLA) on innate immune monocytes. Class II HLA proteins present peptide antigens for recognition by and activation of antigen-specific T lymphocytes. One monocyte class II protein, mHLA-DR, can be measured by flow cytometry. Downregulated mHLA-DR indicates reduced monocyte responsiveness, as measured by *ex-vivo* cytokine production in response to endotoxin stimulation. Our literature survey reveals low mHLA-DR expression on peripheral blood monocytes correlates with increased risks for infection and death. For mHLA-DR, 15,000 antibodies/cell appears clinically acceptable as the lower limit of immunocompetence. Values less than 15,000 antibodies/cell are correlated with sepsis severity; and values at or less than 8000 antibodies/cell are identified as severe immunoparalysis. Several experimental immunotherapies have been evaluated for reversal of sepsis immunoparalysis. In particular, sargramostim, a recombinant human granulocyte-macrophage colony-stimulating factor (rhu GM-CSF), has demonstrated clinical benefit by reducing hospitalization duration and lowering secondary infection risk. Lowered infection risk correlates with increased mHLA-DR expression on peripheral blood monocytes in these patients. Although mHLA-DR has shown promising utility for identifying sepsis immunoparalysis, absence of a standardized, analytically validated method has thus far prevented widespread adoption. A clinically useful approach for patient inclusion and identification of clinically correlated output parameters could address the persistent high unmet medical need for effective targeted therapies in sepsis.

## Introduction

Sepsis, a heterogeneous clinical syndrome, reflects a pathophysiologic state of robust systemic inflammatory response, typically to infection (1–4). This inflammatory response leads to biochemical and physiologic abnormalities that in some patients progress to multiple organ dysfunction syndrome (MODS) and death. Sepsis outcomes have improved over time with advances in antibiotic therapy, fluid/pressor therapy, and dysfunctional organ support. Although most patients recover, sepsis remains a primary cause of intensive care unit (ICU) deaths with mortality at about 26% (1, 5, 6). In the United States (US), an estimated 1.7 million adult sepsis cases are diagnosed annually, leading to more than 350,000 deaths each year (7). Globally, 49 million sepsis cases in 2017 led to 11 million deaths (8). Incidence is highest in the elderly and very young. With high morbidity, mortality, and associated costs, sepsis remains a serious, life-threatening disease with persistent high unmet medical need (9).

Clinical sepsis typically presents with fever, low blood pressure, elevated heart rate, and elevated white cell count (3, 10, 11). While these signs are non-specific, they result from systemic innate immune cell activation due to infectious agents (bacterial, viral, or fungal) or noninfectious etiologies, such as: trauma; burns; surgery; pancreatitis; and cardiac, kidney, or liver injury (1, 4). Regardless of underlying cause, sepsis progression can lead to shock, organ dysfunction, and death (3, 12, 13). In this setting, a constellation of findings support diagnosis, including: clinical, lab, radiologic, physiologic, and microbiologic data (10, 11). Nonetheless, knowledge around sepsis and septic shock continues to advance as we learn more about immunological interactions of innate and adaptive immune responses to infection (10, 11, 14–17).

Over recent decades, molecular and cellular studies have sought to categorize sepsis into endotypes that stratify patient risk and identify therapeutic options (18). While antimicrobial therapy is recommended for all patients with sepsis, level of supportive care varies for those with mild *vs* severe sepsis (19–21). For patients with mild sepsis, fluid therapy, metabolic support, and corticosteroids may be sufficient. In severe sepsis, organ dysfunction necessitates additional supportive care, such as ventilation, vasopressors, and blood product transfusions. Identification of patient subsets might enable effective targeting of new therapies, either to inhibit a disease driver or to correct a deficiency (22). Similarly, selection of patients with elevated risk based on host characteristics or responses might enable targeting study therapies to those in greatest need (23). Despite progress in identifying sepsis endotypes, challenges persist in their clinical validation, as well as their implementation to improve outcomes (24, 25).

## Sepsis immunoparalysis

One prominent model of sepsis pathophysiology describes 2 opposing states of immune dysregulation (26). In this model, a systemic inflammatory response syndrome (SIRS) induces a subsequent compensatory anti-inflammatory response syndrome (CARS). CARS is associated with an increased risk for secondary infections, shock, and organ dysfunction and increased mortality (1, 3, 27). While CARS is clinically occult, hyporesponsive innate and adaptive immune cells have been identified (3, 4, 28).

Severe CARS is also known as immunoparalysis (IP) (3, 26). Sepsis IP has been described to feature dysfunctional monocytes, immune cell depletion, and emergence of regulatory T cells (1, 29, 30). Also, sepsis IP associates with MODS, nosocomial infections, longer ICU hospitalization, and increased mortality (3, 4, 26, 29, 31–33). Notably, MODS comprises impaired function in multiple visceral organs and is associated with high mortality (34).

Despite potential validity and utility of markers for sepsis IP, such as human leukocyte antigen-DR (HLA-DR), tumor necrosis factor (TNF)-α, or absolute lymphocyte counts (ALC), the CARS paradigm faces 2 fundamental challenges (3, 4, 28, 35). First, compensatory molecular or cellular anti-inflammatory mechanisms by which immune cells become hyporesponsive in CARS remain undefined. Second, no diagnostic criteria exist to identify CARS. Rather, tests for immunosuppression/IP focus on immune cell dysfunction alone, independently of causation (35).

We propose here a biologic model of sepsis IP. This model combines recent observations in myeloid cell biology with key features of sepsis immunology (3, 26, 31). In addition, it provides rationale for therapeutic use of sargramostim (Leukine^®^), a yeast-derived, glycosylated recombinant human (rhu) granulocyte-macrophage colony-stimulating factor (GM-CSF).

### Proposed mechanism of sepsis IP

Mononuclear phagocytes (MNPs) include circulating blood monocytes, dispersed tissue-bound macrophages, and dendritic cells (DCs) that may be either circulating or tissue-bound (36). While macrophages may live for years, blood monocytes have a circulating half-life of only 2 to 3 days (36, 37). Also, while circulating monocytes can replace tissue-resident macrophages, turnover rate varies by organ system. Turnover is higher in barrier organs—for example, gut and dermis—than in other organs, such as heart, pancreas, liver, and central nervous system. Replacement may be hastened in any organ by a local inflammatory process that leads to monocyte influx.

Innate immune responses act rapidly as a first line of defense against invasive, infectious pathogens (1). Initially, neutrophils and monocytes recognize pathogen-associated molecular patterns (PAMPs) and damage-associated molecular patterns (DAMPs). These interactions induce MNPs to release multiple cytokines, such as TNF-α, interleukin (IL)-1, and IL-6, that attract and activate other immune cells (1, 4). While neutrophils primarily kill microbes, MNPs kill microbes and, in addition, present their unique antigenic content to the adaptive immune system (4, 38).

**Figure 1:**
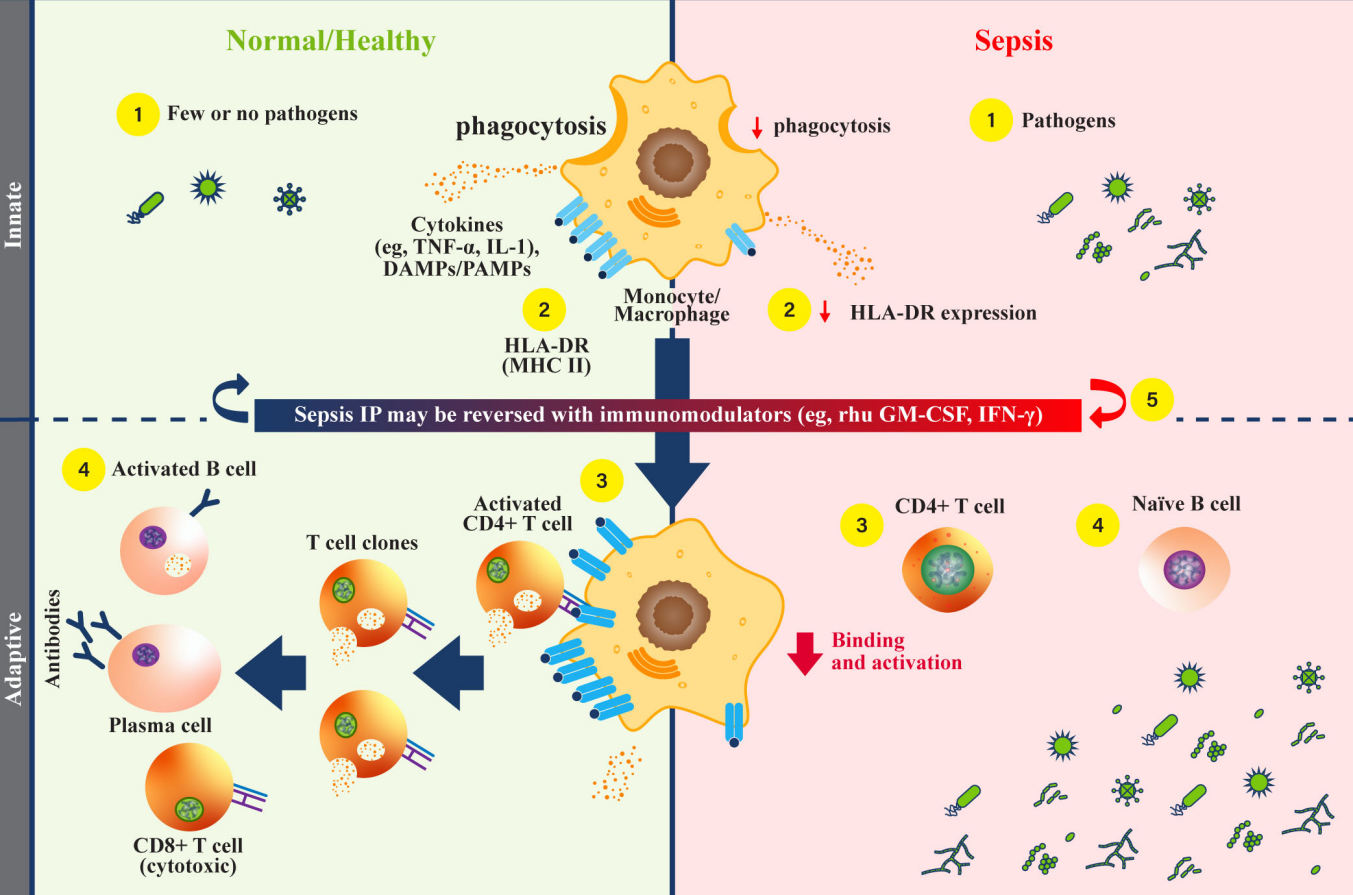
Monocytes and HLA-DR function during sepsis. **(Normal/Healthy; left)** (1) Innate immune cells respond to infectious pathogens by phagocytosis, cytokine secretion, and antigen presentation (1, 4). (2) Phagocytosed pathogens are broken down, then combined as peptides with class II major histocompatibility complex (MHC) (e.g., human leukocyte antigen-DR isotype [HLA-DR], and localized to the cell surface) (4, 40). (3) Peptide-MHC complexes on antigen presenting cells engage with CD4+ helper T cells to activate an adaptive immune response, triggering cytokine release (4, 40). (4) Activated CD4+ T helper cells undergo clonal expansion, activate CD8+ T cells, and mediate B cell activation (1, 26, 41). Activated B cells then differentiate into plasma cells that secrete antibodies, comprising a humoral response. **(Sepsis IP; right)** (1) Dysfunctional monocytes/macrophages demonstrate reduced pathogen phagocytosis, reduced antigen presentation, and variable cytokine profiles (4). (2) Dysfunctional monocytes/macrophages express less antigen-bound HLA-DR proteins, leading to reduced engagement with the adaptive immune system (4). (3) Without effective antigen presentation by monocytes/macrophages, CD4+ T cells are not activated, and adaptive immune responses are rendered ineffective in clearing pathogens (1). (4) Naïve B cells are not activated by CD4+ T cells, and antibody producing plasma cells are not generated. With an inadequate humoral immune response, pathogens survive and replicate (1, 42). (5) Recombinant human (rhu) granulocyte-macrophage colony-stimulating factor (GM-CSF) may restore monocyte/macrophage function (4). DAMP, damage-associated molecular patterns; GM-CSF, granulocyte-macrophage colony-stimulating factor; HLA-DR, human leukocyte antigen-DR isotype; IFN, interferon; IL, interleukin; IP, immunoparalysis; PAMP, pathogen-associated molecular patterns; MHC II, class II major histocompatibility complex; rhu, recombinant human; TNF, tumor necrosis factor.

MNPs link innate and adaptive immune systems by their ability to adopt either pro- or anti-inflammatory functions (4, 39). Pro-inflammatory functions eliminate infectious or injurious stimuli and activate antigen-specific helper T lymphocytes, whereas anti-inflammatory functions maintain homeostasis, conduct efferocytosis, and thereby control autoimmunity. Critically, MNPs express class II major histocompatibility complex (MHC) proteins that activate antigen-specific helper T lymphocytes and secrete cytokines to nourish and/or activate diverse cell types (**Figure 1**). More numerous neutrophils by contrast are primarily pro-inflammatory, live only for days after a 6- to 12-hour circulating half-life, and do not characteristically present foreign antigens to adaptive immune lymphocytes (43).

The adaptive immune system comprises antigen-specific T lymphocytes that are cytotoxic, or are responsible for self-tolerance (T regulatory cells) as well as antibody-producing plasma cells that result from B cell differentiation (1, 3). Although initially slower to respond than the innate immune system, the adaptive immune system drives antigen-specific recognition and generates immunologic memory. Immunologic memory generates faster, stronger repeat immune responses against previously encountered antigens.

In sepsis, both innate and adaptive arms of the human immune system are altered (1). In addition, multiple cytokine levels are elevated, including GM-CSF. These cytokines drive proliferation of circulating innate immune cells, including neutrophils, monocytes, and eosinophils, by signaling through specific cell surface receptors (44, 45). For example, high affinity GM-CSF receptors are found principally on myeloid cells, including neutrophils, MNPs, and eosinophils.

Numerous cytokines, including GM-CSF, have pleiotropic effects that vary depending on local cytokine concentrations in the vicinity of specific cell surface receptors (44, 46–48). GM-CSF pleiotropism relies on higher order extracellular assembly of heterodimeric receptor chains, as well as 4 distinct intracellular signaling pathways, including: mitogen activated protein kinase (MAPK); nuclear factor kappa-B cells (NFĸB); phosphoinositide 3-kinase (PI3K); and signal transducer and activator of transcription 5 (STAT5). Such diversity explains GM-CSF’s capacity to generate survival, differentiation, activation, and/or proliferation signals, depending on cytokine concentration at the receptor level, as well as other local stimuli. At low GM-CSF concentrations, PI3K signaling leads to survival, whereas at high concentrations, PI3K, MAPK, and STAT5 signaling lead to survival and cell proliferation (47, 48). Correspondingly, both ligand and dose-specific effects on NFĸB signaling have been described in primary macrophages (49, 50), and such effects have been observed to influence epigenomic programming (51).

Recently, GM-CSF effects on MNP metabolism were revealed in mouse models with disrupted GM-CSF signaling (52). These models demonstrated a critical role of GM-CSF in maintaining mitochondrial structure and function, as well as fatty acid beta oxidation, tricarboxylic acid cycle activity, oxidative phosphorylation, and adenosine triphosphate (ATP) generation. These effects of GM-CSF on metabolic capacity enable MNPs to fulfill energy-intensive innate immune functions, including: respiratory burst generation, phagocytosis, antigen presentation, cytokine secretion, and efferocytosis (52–54). All these functions rely on metabolic energy and fail in its absence. By extension, metabolic capacity in tissue-bound macrophages throughout the body may be maintained by ongoing low-level and/or pulsatile GM-CSF expression. This activity aligns with known ongoing low-level yet plastic GM-CSF expression by diverse cell types, including endothelial, epithelial, and immune cells, as well as fibroblasts (2, 55).

We hypothesize that myeloid proliferation driven by high cytokine levels in sepsis leads to cell division that outpaces time and/or GM-CSF stimulation needed for maturation of cellular metabolic capacity. Thus, sustained high inflammatory cytokine secretion may counterintuitively result in degradation of metabolic capacity of newly formed MNPs to fulfill immune functions. Consequently, immature MNPs with insufficient metabolic capacity to support normal innate immune functions appear “immunosuppressive.” In support of this model, GM-CSF reverses monocyte hyporesponsiveness in multiple *in-vitro* systems (56–60). Multiple reports support that GM-CSF increases blood monocyte levels, upregulates monocyte responsiveness, and increases HLA-DR expression, which is known to enhance antigen presentation and adaptive immune responses (48, 54, 61, 62).

## Immune biomarkers in sepsis IP

Numerous immune biomarkers have been assessed to seek prognostic and/or predictive markers for patient stratification and therapy in sepsis (12). Methods studied include: neutrophil respiratory burst in response to pathogen exposure; lymphocyte and monocyte counts; neutrophil-to-lymphocyte and monocyte-to-lymphocyte ratios; monocyte programmed death-ligand 1 (PD-L1) expression; IL-10; and transcriptomics, among others (26, 63–67). Most such methods have not been widely adopted due to challenges in analytic validity, clinical validity, and/or clinical utility. Methods with evidence of clinical validity include HLA-DR quantitation of blood monocytes, TNF-α release from peripheral blood cells after *ex-vivo* lipopolysaccharide (LPS) stimulation, and ALC (29, 30). Biological rationale, validation challenges, and clinical data for each of these 3 markers are summarized below.

### Blood monocyte HLA-DR expression

The polymorphic MHC gene family in humans is on chromosome 6 and encodes multiple class II MHC proteins, including HLA-DP, HLA-DQ, and HLA-DR (68). Historically, these proteins were recognized as transplantation antigens, serving as targets for immune rejection of transplanted tissue. During infection, MNPs phagocytose pathogens that are then digested to yield foreign peptides that combine intracellularly with class II MHC proteins, such as HLA-DR (4, 69). Normally, monocytes and macrophages express HLA-DR levels ranging from 15,000 antibodies bound per cell (Ab/c) to as high as 60,000 Ab/c (70, 71); and a commonly used lower limit of HLA-DR in healthy subjects is 15,000 Ab/c (33, 72, 73). The large spread in the reported HLA-DR levels is most likely explained by biologic variability, as well as differences in assay reagents and flow cytometry methods used over years to quantitate HLA-DR expression levels (74–77). Peptide-MHC complexes are transported to the cell surface where they mediate antigen-specific recognition by CD4+ helper T lymphocytes. Once activated by peptide-MHC recognition, CD4+ T lymphocytes boost adaptive immune responses by activating other T and B lymphocytes that can recognize and target the invading pathogen (3, 4, 26). Because HLA-DR functions as the bridge between innate MNPs and antigen-specific T lymphocytes, low HLA-DR levels lead to diminished antigen presentation and reduced adaptive immune activation (4, 78). When HLA-DR is low, CD4+ T lymphocytes are not activated, hence cannot augment either B-cell stimulation to produce specific antibodies or CD8+ cytotoxic T lymphocyte generation to target infected cells directly (3, 4).

Despite HLA protein diversity, common determinants recognized by monoclonal antibodies enable flow cytometric quantitation of surface class II MHC expression level on blood cells (4, 40, 76). Although flow cytometry enables monocyte HLA-DR (mHLA-DR) quantitation, other cells expressing HLA-DR are also detected, including DCs, macrophages, B cells, and T cells (4, 30, 76, 79–82). Thus, to generate mHLA-DR specificity, cells are also stained for CD14 (also known as the LPS receptor), of which, expression is restricted to monocytes. Combined CD14 and HLA-DR staining enables quantitation of CD14+ classical and intermediate monocytes, the most abundant and rapidly replenished populations in blood. Results are typically reported either as percent of CD14+ monocytes expressing HLA-DR or as mean fluorescence intensity (MFI) of antibody against HLA-DR on CD14+ monocytes (77, 83).

HLA-DR downregulation and reduced monocyte responsiveness are described features of sepsis IP (4, 26). As detailed in **Table 1**, low HLA-DR correlates with adverse clinical outcomes, including increased risk for nosocomial infections, end-organ failure, longer ICU hospitalizations, and mortality (30, 33, 75, 84–89, 91–93).

**Table 1:** Studies connecting monocyte HLA-DR to clinical outcomes.

Study	Condition	Monocyte Function (Test)	mHLA-DR Monitoring	Results	Clinical Implications
**Prospective, single center, observational study (n=1053)** (84)	Sepsis	mHLA-DR Ab/c (FC)	Sample 1 collected and analyzed within 3 days of ICU admission; sample 2 collected and analyzed within first week	• Low mHLA-DR expression at presentation associated with initial disease severity assessment (R2 = 0.28; p<0.01) • Persistence of a low mHLA-DR (< 8000 Ab/c), measured between Day 5 and Day 7, was associated with a later occurrence of IAIs (p=0.01)	Higher IAI risk associated with persistent low mHLA-DR measure
**Prospective, single center, observational study (n=51)** (85)	Cardiac arrest	mHLA-DR MFI (FC)	Samples collected at 12, 24, and 48 hours after cardiac arrest	• In patients following cardiac arrest and cardiopulmonary resuscitation, downregulation of HLA-DR expression was observed mainly in classical monocytes and correlated with norepinephrine dose	No correlation between mHLA-DR expression and 30-day mortality
**Prospective, single center, observational study (n=36)** (86)	Trauma	mHLA-DR Ab/c (FC)	Periodic monitoring; samples collected and analyzed at Days 1, 3, and 8 after injury	• 22% of patients had secondary infections, all of which had HLA-DR < 15,000 Ab/c at Days 3–4 • Not powered to establish an association between HLA-DR and secondary infections (p=0.22)	Trend for secondary infections with low mHLA-DR levels at Day 3
***Post-hoc* analysis of ETASS Study (n=273)** (87)	Sepsis	%mHLA-DR+ (FC)	Single measurement; early immune status evaluated by the %mHLA-DR in total monocytes within 48 hours after onset of sepsis	• Patient classified as IP when mHLA-DR ≤ 30% and non-IP when > 30% mHLA-DR • Higher mortality rate for elderly with IP *vs* elderly without IP (53.4% *vs* 32.5%; p=0.009) • For non-elderly patients, no difference in mortality rates for IP *vs* non-IP (33.5% *vs* 26.0%; p=0.541)	Higher hospital and ICU mortality risk associated with low mHLA-DR measure for elderly
**Prospective, observational study (n=24)** (88)	Critically ill, COVID-19	mHLA-DR Ab/c (FC)	Periodic monitoring	• Lower mHLA-DR expression for COVID-19 *vs* healthy subjects (11,860 Ab/c *vs* 15,000–45,000 Ab/c; p-value not reported) • Higher mHLA-DR expression for COVID-19 *vs* sepsis (bacterial infections were the drivers of sepsis (75); 11,860 Ab/c *vs* 5211 Ab/c; p<0.0001)	mHLA-DR expression kinetics revealed no change over time. No secondary infections were observed during the follow-up period for patients with COVID-19
**Prospective, observational study (n=241)** (75)	Sepsis	mHLA-DR Ab/c (FC)	Periodic monitoring; samples collected and analyzed at 3 time points (Day 1 or 2; Day 3 or 4; Day 6, 7, or 8)	• No difference in mHLA-DR expression between pathogen categories (e.g., Gram-positive, Gram-negative) and sites of infection (e.g., abdominal, respiratory tract, urinary tract) • Greater increase in mHLA-DR expression for survivors *vs* non-survivors (AUROC, 0.65; p=0.01)	Increased risk of secondary infections and 28-day mortality associated with declining mHLA-DR expression
**Retrospective, observational study (n=297)** (89)	Sepsis	%mHLA-DR+ (FC)	Periodic monitoring; samples collected at Days 1, 3, and 7 after hospital admission	• Lower %mHLA-DR+ expression on Day 3 for patients with secondary infections *vs* those without secondary infections (28.6% *vs* 41.1%; p=0.048) • Higher in-hospital (45.7% *vs* 25.4%; OR, 2.472; p=0.001), 30-day (34.8% *vs* 23.4%; OR, 1.744; p=0.041), and 90-day mortality rates (42.4% *vs* 25.4%; OR, 2.165; p=0.003) for patients with secondary infections *vs* those without secondary infections	Increased risk of secondary infections associated with lower mHLA-DR expression
**Prospective, single center, observational study (n=56)** (90)	Sepsis	mHLA-DR Ab/c (FC)	Periodic monitoring; samples collected and analyzed at Days 1, 3, and 7 after injury	• Lower levels of mHLA-DR (5913–7927 Ab/c *vs* 25,477–34,295 Ab/c; p<0.001) and lower CD4+ T cells (332–1186 cells/µL *vs* 895–2187 cells/µL; p<0.01) for patients with septic shock *vs* healthy controls • Lower levels of mHLA-DR for those with secondary infection *vs* those without secondary infection (Days 1–2 mHLA-DR, 4146 Ab/c *vs* 8704 Ab/c; p=0.28; Days 3–5, 4398 Ab/c *vs* 8474 Ab/c; p=0.022) (91)	Increased secondary infections associated with lower mHLA-DR expression
**Prospective, controlled study (n=74)** (92)	Critically ill (including sepsis; n=12)	%mHLA-DR+ and mHLA-DR MFI (FC)	Daily monitoring; samples collected and analyzed Days 1–4 of PICU stay	• Lower mHLA-DR expression (67% *vs* 95%; p<0.001) and lower HLA-DR MFI within monocyte subsets (3219 *vs* 6545; p<0.001) for critically ill children *vs* controls	Increased nosocomial infection risk with lower mHLA-DR expression on classical monocytes
**Prospective, single center, *ex-vivo* study (n=19)** (30)	Sepsis	mHLA-DR MFI (FC) and HLA-DR mRNA (PCR)	Single measurement; samples collected and analyzed on Day 1 of inclusion	• Higher monocyte numbers in peripheral blood (p<0.001) but lower HLA-DR MFI (p<0.001) and mRNA HLA-DR levels (p<0.001) for patients with sepsis *vs* controls	Higher 28-day mortality rate associated with low HLA-DR
**Prospective, observational study (n=100)** (33)	Trauma	ΔmHLA-DR (FC)	Periodic monitoring; samples collected every 2 days; subsequent samples after Day 5 were not presented (occurred after sepsis development)	• mHLA-DR has predictive potential for development of sepsis after major trauma: • Slope of mHLA-DR expression between Days 3–4 and Days 1–2 (OR, 9.0; p=0.0009)	Higher risk for sepsis development with greater reduction of mHLA-DR levels between Days 1–2 and Days 3–4
**Prospective, observational study (n=79)** (93)	Sepsis	ΔmHLA-DR (FC)	Periodic monitoring; samples collected and analyzed between Days 0, 3, and 7 after injury	• Greater ΔmHLA-DR from Day 0 to Day 7 for survivors *vs* non-survivors (16.9 *vs* 4.55; p=0.038) • Smaller ΔmHLA-DR from Day 0 were associated with higher 28-day mortality (ΔmHLA-DR, Days 0–3 ≤ 4.8%; OR, 94.71; p<0.001; ΔmHLA-DR, Days 0–7 ≤ 9%; OR, 51.04; p<0.001)	Higher 28-day mortality associated with smaller ΔmHLA-DR over 7 days

ΔmHLA-DR, change in monocyte human leukocyte antigen-DR; %mHLA-DR+, percent of monocytes positive for human leukocyte antigen-DR; Ab/c, antibodies bound per cell; AUROC, area under receiver operating curve; COVID-19, coronavirus disease of 2019; ETASS, Efficacy of Thymosin Alpha 1 for Severe Sepsis; FC, flow cytometry; IAI, intensive care unit-associated infections; ICU, intensive care unit; IP, immunoparalysis; MFI, mean fluorescence intensity; mHLA-DR, monocyte human leukocyte antigen-DR; mRNA, messenger ribonucleic acid; OR, odds ratio; PCR, polymerase chain reaction; PICU, pediatric intensive care unit.

Inter-laboratory variability initially posed a challenge to analytic validity of HLA-DR testing to identify sepsis IP (4). Now, a system offering standardized quantitative measurement of cell surface HLA-DR proteins (Quantibrite™; Becton, Dickinson and Company [BD]) is available. Developed in 2001, Quantibrite™ beads allow estimation of Ab/c, enabling monocyte cell surface HLA-DR protein quantitation to stratify patients based on mHLA-DR levels (79, 94–96). This assay uses phycoerythrin (PE)-labeled anti-HLA-DR monoclonal antibodies for estimating Ab/c (97). Geometric MFI values can be analyzed further to calculate numbers of Ab/c, which represents numbers of HLA-DR proteins on the monocyte surface (96, 97). Using standard instrument settings, flow cytometry data are converted into number of PE molecules per cell. Based on a known ratio of PE to antibodies against HLA-DR, Ab/c can be calculated, hence quantitating HLA-DR protein on CD14+ monocytes. With Quantibrite™, moderate immunosuppression is defined as about 10,000–15,000 Ab/c (74, 79). In several studies, a cut-off value of 8000 Ab/c was used to indicate IP. HLA-DR levels below 8000 Ab/c indicate more severe sepsis IP (4, 79). In some studies, 30% CD14+/HLA-DR+ cells corresponded to 5000 Ab/c for severe IP, whereas 45% CD14+/HLA-DR+ cells corresponded to about 8000 Ab/c for moderate IP (4, 79). Numerous studies have employed Quantibrite™ to measure HLA-DR-defined IP (26, 33, 70, 73–75, 77, 88, 90, 98–101).

Multiple literature analyses support mHLA-DR expression by flow cytometry as a sepsis IP biomarker and mortality predictor (80). One such review evaluated mHLA-DR in patients with complicated intra-abdominal infections and sepsis from 12 studies (n=761) (102). Results from 10 of these studies showed strong associations between low mHLA-DR expression and mortality. By contrast, 2 studies showed no prognostic value of mHLA-DR expression level. Proposed factors contributing to nonsignificant results in these 2 studies include: homogeneity of enrolled patients, young age, small sample sizes, and heterogeneity among experimental protocols (77, 87, 100, 103). Another review assessed mHLA-DR in critically ill patients with coronavirus disease of 2019 (COVID-19), sepsis, or bacterial infections from 15 studies (n=1160) (104). Of these studies, 4 monitored mHLA-DR expression with flow cytometry by a standardized protocol that reported results as Ab/c. Initial mHLA-DR expression was lower for COVID-19 patients than for controls (10,000 Ab/c *vs* 15,000 Ab/c) yet higher for COVID-19 patients than for septic shock patients (10,000 Ab/c *vs* 5000 Ab/c). Lower mHLA-DR expression was associated with higher ICU mortality and greater disease severity at hospital admission. A meta-analysis evaluated 8 prospective cohort studies to evaluate HLA-DR as a biomarker for sepsis in patients after trauma (n=639) (105). Results from 7 studies showed that HLA-DR by flow cytometry for detecting sepsis IP had a pooled sensitivity of 81% and a pooled specificity of 67%.

While various thresholds for detecting IP have been proposed, a minimum threshold for raising secondary infection and mortality risks has to date been neither standardized nor adopted (3, 4, 77, 84, 106). Hence, HLA-DR testing by flow cytometry can now be implemented with analytic validity, and multiple studies support its clinical validity. Yet, both a definitive threshold for sepsis IP and clinical utility for therapeutic response prediction remain, for now, unconfirmed.

Notably, 3 additional approaches to mHLA-DR measurement have been investigated. First, measurement of HLA-DR expression levels by polymerase chain reaction (PCR) was explored in several clinical studies (4, 70, 80). In 1 such study, quantitative real-time PCR (qRT-PCR) and mHLA-DR flow cytometry were used to assess HLA-DR and class II transactivator (CIITA) in patients with bacteremic sepsis (n=60) (70). TaqMan gene qRT-PCR expression assays were used to measure HLA-DR-α subunit (HLA-DRA) and CIITA, whereas Quantibrite™ was used to measure mHLA-DR by flow cytometry. Similar patterns for initial reductions in HLA-DRA, mHLA-DR, and CIITA were all followed by subsequent increases over time (p<0.001). Hence, qRT-PCR yields results somewhat similar to flow cytometry with low variability and reproducibility. While qRT-PCR may be robust for detecting HLA-DR expression in patients with sepsis, qRT-PCR results are non-specific for monocytes since circulating DCs, B cells, and activated T cells also express HLA-DR (70, 80). As such, it may not reliably reflect mHLA-DR expression in monocytes that drives sepsis IP (4, 80, 107).

Second, myeloid-derived suppressor cells (MDSCs) have been described in patients with sepsis (4, 108–110). Although not standardized, all MDSC descriptions include “low HLA-DR expression.” Hence, MDSCs are invariably monocytes with low HLA-DR. In sepsis, MDSCs associate with: prolonged immunosuppression, diminished T cell functions, development of nosocomial infections, higher reinfection rates, and hospital readmissions (4, 109, 111, 112).

Finally, several studies support that dynamic changes by serial mHLA-DR monitoring might predict mortality better than static mHLA-DR monitoring (80, 113, 114). Correspondingly, persistence of low mHLA-DR levels suggests slow or no recovery from sepsis IP (4, 12, 13, 113, 115, 116). Given inter-individual variability of mHLA-DR in sepsis, dynamic change or HLA-DR slope might increase prognostic significance of low mHLA-DR expression for mortality prediction (4, 13, 93, 98, 117). Thus far, no standardized approaches to serial mHLA-DR monitoring have been either developed or tested prospectively.

### Pediatric *vs* adult mHLA-DR

As in adults, low mHLA-DR in children associates with nosocomial infections and mortality (92, 118–121). Nonetheless, patient age affects monocyte subtypes and function, so direct comparison of adults *vs* children may be confounding (121). While adult monocytes are predominantly classical (CD14+/CD16-), neonatal monocytes are mostly intermediate (CD14+/CD16+) or nonclassical (CD14-lo/CD16+) subtypes that express lower levels of HLA-DR (121–123). These differences result in reduced T cell activation in neonates compared with adults (121). Also, neonates have proportionally more regulatory T cells than adults, and that difference may also limit immune responses in children with sepsis (124, 125).

One study compared mHLA-DR expression among critically ill children with sepsis, trauma-related hospital acquired infection, or recent surgery (n=37; median age, 9 years) *vs* healthy control children (n=37; median age, 3 years) (92). Results showed lower mHLA-DR expression (67% *vs* 95%; p<0.001) and lower mHLA-DR MFI (3219 *vs* 6545; p<0.001) for critically ill children *vs* healthy controls at all examined time points, in particular on classical monocytes and in children admitted for sepsis. Another study evaluated blood samples in hospitalized children with sepsis (n=30) *vs* healthy controls (n=21) for mHLA-DR expression using Quantibrite™ technology (98). As with adults, mHLA-DR expression in pediatric patients with sepsis was lower than that in controls (p=0.0001). Finally, a prospective, single-center, observational study evaluated mHLA-DR levels using Quantibrite™ in children with septic shock admitted to a pediatric ICU (n=26; median age, 2 years) with healthy controls (n=30) (90). As seen elsewhere, mHLA-DR levels were lower for patients with septic shock than for healthy controls (p<0.001).

### *Ex-vivo* blood cell TNF-α secretion

While HLA-DR is well-documented for sepsis IP detection, other potential biomarkers are also being explored. LPS-induced TNF-α production from peripheral blood cells reflects innate immune system function *via* myeloid cell capacity to respond to an inflammatory stimulus (3, 30, 126). Although both *ex-vivo* TNF-α secretion and HLA-DR expression assess monocyte dysfunction *via* metabolic capacity to fulfill basic immune functions, *ex-vivo* TNF-α secretion is less specific for monocytes as responding myeloid cells include both neutrophils and monocytes. Independent of sepsis IP, TNF-α levels may also be influenced by a variety of other factors, such as: type of LPS used, blood volume, incubation conditions, and LPS concentration (3).

In contrast to substantial literature examining mHLA-DR prognostic significance in adult sepsis, there are fewer reports on TNF-α, and most are in small groups of children (3, 74, 91, 127, 128). Overall, these studies support clinical validity of measuring TNF-α by *ex-vivo* LPS stimulation. Although a few studies describe standardized protocols for measuring LPS-induced TNF-α production for sepsis, scalable analytic validity may remain challenging (29, 129). As seen for HLA-DR quantitation, no receiver operating characteristic (ROC) curve analysis has been performed to define a TNF-α threshold for sepsis IP.

### Absolute lymphocyte count

ALC is another laboratory parameter that reflects immune system function (29, 130). The reference range for ALC varies with age. Normal for adults varies between 1000 and 4800 cells/µL, and for children, between 3000 and 9500 cells/µL (131, 132). Lymphopenia occurs when a patient’s ALC is below normal and can increase risk for infection (133).

In sepsis IP, circulating lymphocyte populations (e.g., CD4+ T cells, CD8+ T cells, B cells) are characteristically reduced due to tissue sequestration and apoptosis (26). Reductions at sepsis onset typically persist for up to 28 days. Increased apoptosis of both innate immune cells and adaptive immune cells in sepsis results in leukopenia, which associates with higher risks of secondary infections and death (134–136).

A retrospective, single-center cohort study monitored blood parameters in patients with bacteremia and sepsis (n=335) for secondary infection risk and mortality (130). Results showed higher ALC at Day 4 for survivors *vs* non-survivors (1100 cells/µL *vs* 700 cells/µL; p<0.0001). Also, 28-day and 1-year mortality were higher in severe (40% *vs* 10% and 58% *vs* 29%; p<0.001) and moderate (25% *vs* 10%; p=0.003, and 40% *vs* 29%; p=0.025) lymphopenia *vs* those without persistent lymphopenia. Multivariable analysis showed that Day 4 ALC was associated with both 28-day (odds ratio [OR], 0.68; p=0.009) and 1-year mortality (OR, 0.74; p=0.008). Severe persistent lymphopenia (< 0.6 x 10^3^ cells/μL) was also associated with development of secondary infections (OR, 2.11; 95% confidence interval [CI], 1.02–4.39; p=0.04) (26, 130). Thus, persistent lymphopenia on the fourth day after a sepsis diagnosis predicted mortality and may be a valid marker of sepsis-induced immunosuppression.

In another single-center study, cross-sectional analysis was performed of ALC as an outcome predictor in patients with sepsis presenting to an emergency department (n=124) (137). Results showed a higher need for ICU admission (51.9% *vs* 14%; p<0.001) and higher rates of 28-day mortality (88.1% *vs* 11.9%; p<0.001) for patients with lymphopenia *vs* those without lymphopenia. In addition, age and sequential organ failure assessment (SOFA) scores were higher for patients with lymphopenia *vs* without.

Lower monocyte counts are also seen in sepsis and can impact health outcomes (64). A retrospective, single-center database analysis of patients with sepsis (n=2012) showed higher 28-day mortality rates, higher bacteremia rates, and higher incidence of organ dysfunction for patients with initial monocyte counts < 250 cells/μL.

## Comparison of HLA-DR, TNF-α secretion, and ALC

Pros and cons of mHLA-DR expression, TNF-α secretion, and ALC as prognostic indicators in sepsis IP are summarized in **Table 2** (4, 26, 27, 30, 35, 130, 138, 139). mHLA-DR expression and TNF-α responsiveness seek to measure similar biology of innate immune MNP dysfunction (26, 27, 130). Correspondingly, in an *ex-vivo* study using blood samples from patients with sepsis or septic shock (n=20), mHLA-DR expression correlated with TNF-α response (30). By contrast, ALC reflects distinct, complementary biology of deficient adaptive immune responsiveness (66).

**Table 2:** Pros and cons of immune biomarkers for sepsis IP (4, 26, 27, 30, 35, 74, 130, 138, 139).

Biomarker	Analysis Method	Pros	Cons
**mHLA-DR**	Flow cytometry	• Reflects monocyte state • Specific to classical monocytes • High analytic validity with BD Quantibrite™ technology	• Requires flow cytometry at or near point of sample collection or expedited shipping to a flow cytometry laboratory • Time sensitive analysis post-sample collection • Sample stability
**TNF-α**	ELISA	• Reflects monocyte state	• Neutrophils also responsive, thus the level of TNF-α is not specific for monocytes • Includes multiple steps • Cell culture, incubation, and centrifugation to isolate supernatants at or near point of sample collection • Analytic validity could be confounded by variation in LPS source
**Lymphocyte**	ALC	• Routinely available	• No differentiation among types of lymphocytes • Thresholds undefined

ALC, absolute lymphocyte count; BD, Becton, Dickinson and Company; ELISA, enzyme-linked immunosorbent assay; LPS, lipopolysaccharide; mHLA-DR, monocyte human leukocyte antigen-DR; TNF, tumor necrosis factor.

HLA-DR expression offers acceptable analytic validity based on well characterized monoclonal antibodies and Quantibrite™ technology (74). Nonetheless, testing for this biomarker requires flow cytometry of fresh or stabilized cells, necessitating either shipping to a central facility or timely local analysis (74, 140). By contrast, TNF-α secretion requires local site addition of LPS to blood samples and incubation followed by analysis of frozen cell supernatants by enzyme-linked immunosorbent assay (ELISA) (138). This procedure generates need for trained site staff to perform *ex-vivo* LPS stimulation reliably. Notable analytic validity hurdles for TNF-α secretion include variability in LPS source and *ex-vivo* stimulation protocols, as well as non-specificity for monocyte *vs* neutrophil secretion. Neutrophils may be a significant source of TNF-α due to their higher abundance in whole blood relative to monocytes (138, 139, 141). While ALC measurement is logistically simple, inexpensive, and reflects adaptive immune function directly, ALC alone does not directly reflect innate immune function (142). Also, a threshold to define sepsis IP based on ALC remains, to date, undefined (130).

## Immunostimulatory agents in sepsis

Consequences of sepsis IP are severe and contribute to sepsis mortality (26, 29). However, sepsis IP may be reversible since about one third of severe sepsis survivors regain immune function (29). As such, many drug trials have focused on targeting the clinically overt state of SIRS with pharmacologic agents that have anti-inflammatory effects. Though, most such agents have failed to improve outcome, and none has yet been shown to improve survival. Nonetheless, investigation continues of immunostimulatory agents that aim to reverse CARS effects (1, 3).

Experimental immunotherapies for sepsis IP have been shown to decrease ICU stay duration and secondary infection risk (3, 62). Notably, immunostimulating agents have shown promise for reversing IP, including: recombinant IL-7, programmed death 1 (PD-1)/PD-L1-specific antibodies, recombinant interferon (IFN)-γ, and recombinant GM-CSF (3, 12, 29, 62).

IL-7 is a potent anti-apoptotic cytokine required for lymphocyte survival and expansion that has shown potential benefits in patients with sepsis (143). The phase 2 IRIS-7 study evaluated IL-7 at varying frequencies *vs* placebo in patients with septic shock and severe lymphopenia (n=27). At Day 29, results showed higher ALC for IL-7 relative to placebo study therapy (+0.99–1.30 x 10^3^ lymphocytes/µL *vs* 0.99 x 10^3^ lymphocytes/µL; p=0.004). Elevated ALC persisted for 2–4 weeks after discontinuing IL-7.

PD-1 and PD-L1 are upregulated in sepsis and other inflammatory states (including cancer) (144). Clinical responses seen with PD-1/PD-L1 inhibitors in tumors suggested potential benefits for sepsis IP (27, 29), and a phase 1 trial of nivolumab in patients with sepsis (n=31) demonstrated safety. Larger clinical studies, however, were stopped by the sponsor (29, 145).

Pro-inflammatory cytokine IFN-γ plays a role in both innate and adaptive immune responses (146). One trial showed that IFN-γ study treatment restored mHLA-DR expression in patients with sepsis IP (4, 147). A separate, small, randomized, double-blind study (n=18) evaluated recombinant IFN-γ *vs* recombinant GM-CSF *vs* placebo in healthy volunteers given *E. coli* endotoxin. IFN-γ increased mHLA-DR expression and TNF-α levels but did not significantly improve symptom scores (148). In contrast, treatment with GM-CSF showed results trending in the same direction as IFN-γ, but were not statistically significant compared with placebo. Finally, a prospective case series described patients with invasive fungal infections treated with recombinant IFN-γ (n=8) (149). Notably, 5 of these 8 patients were considered to have IP, defined as < 50% HLA-DR+ monocytes. Treatment with recombinant IFN-γ restored immune function as indicated by increased HLA-DR expression in those with IP, increased *ex-vivo* cytokine production (e.g., TNF-α, IL-17, IL-22), and increased total leukocyte counts.

**Figure 2:**
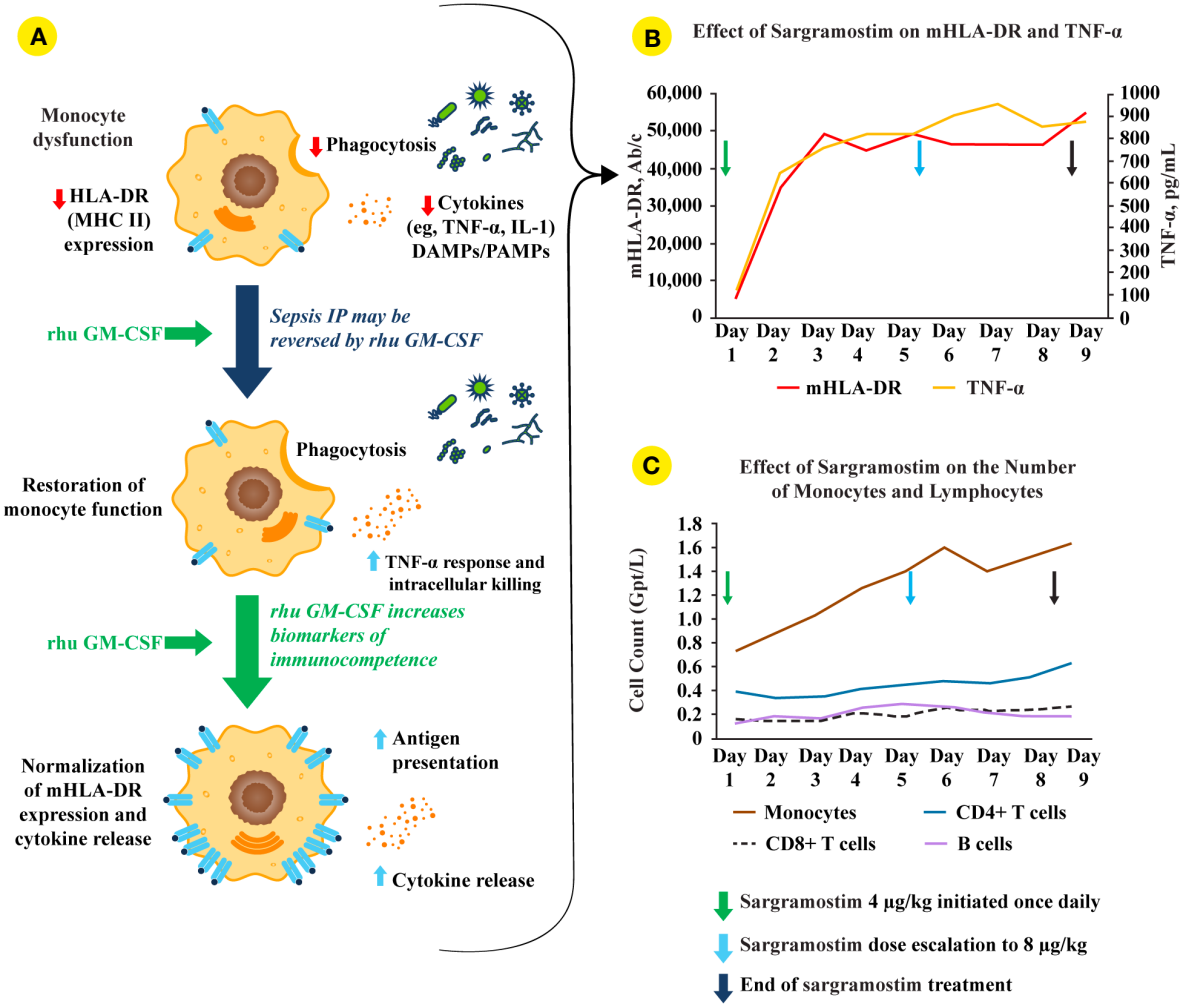
rhu GM-CSF (including sargramostim) stimulates and restores immune function in sepsis IP. **(A; top monocyte)** Impaired monocyte function leads to reduced pro-inflammatory mediator responses, decreased pathogen phagocytosis, and lower human leukocyte antigen-DR isotype (HLA-DR) expression (4). **(A; middle and bottom monocytes)** Treatment with recombinant human (rhu) granulocyte-macrophage colony-stimulating factor (GM-CSF) leads to increased intracellular killing, cytokine secretion, phagocytosis, monocyte (m)HLA-DR expression, and antigen presentation (2, 4). **(B)** In a biomarker-guided study of patients with sepsis IP (n=38), sargramostim was given daily for up to 8 days to patients with sepsis and mHLA-DR lower than 8000 Ab/c (31). Sargramostim treatment led to improved mHLA-DR expression and tumor necrosis factor (TNF)-α responses. **(C)** Sargramostim increased absolute numbers of monocytes and lymphocyte subsets (e.g., CD4+ T cells, CD8+ T cells, B cells) (31). Ab/c, antibody numbers bound per cell; DAMP, damage-associated molecular patterns; GM-CSF, granulocyte-macrophage colony-stimulating factor; HLA-DR, human leukocyte antigen-DR isotype; IL, interleukin; IP, immunoparalysis; MHC II, class II major histocompatibility complex; mHLA-DR, monocyte human leukocyte antigen-DR; PAMP, pathogen-associated molecular patterns; rhu, recombinant human; TNF, tumor necrosis factor.

Therapeutic GM-CSF is available as a rhu protein (sargramostim) that was approved by the US Food and Drug Administration (FDA) in 1991 for myeloid cell reconstitution after cytotoxic chemotherapy (150, 151). Notably, rhu GM-CSF (including sargramostim) augments monocyte metabolic capacity, function, and proliferation (**Figure 2**) (3, 29, 77, 150, 152). In addition, sargramostim has been administered to acutely and critically ill patients, including children across multiple trials (**Table 3**) (31, 99, 126, 148, 153–155). No serious adverse events have been ascribed to sargramostim in these studies, and it did not increase systemic inflammation as measured by pro-inflammatory cytokines (e.g., IL-6 or IL-8). Doses studied were at or below the labeled dose for myeloid reconstitution (250 µg/m^2^/day). In some studies, immune recovery was prompt, within 3 days of sargramostim administration, with trends toward improved infection recovery, reduced hospital stays, and fewer days of mechanical ventilation. Nonetheless, all these studies were underpowered to confirm effects on outcomes. Results of 2 multi-center randomized trials of sargramostim in sepsis IP are also awaited. The ongoing GRACE-2 study (NCT05266001) will evaluate sargramostim *vs* placebo in 400 children with sepsis-induced MODS and IP. Furthermore, mHLA-DR expression will be assessed in this study to establish its clinical utility. In addition, the United Kingdom (UK)-based National Institute for Health and Care Research (NIHR) will sponsor the SepTIC trial that includes investigation of sargramostim for improving outcomes in a high-risk subset of patients admitted to the ICU with sepsis, which is anticipated to begin in mid-2023 (156). Of 3758 adult patients to be enrolled, 1300 with ALC below 1200 cells/µL will be randomized to sargramostim *vs* placebo. The primary endpoint will be 90-day all-cause mortality.

**Table 3:** Use of sargramostim in sepsis to improve clinical outcome and restore normal monocyte function.

Study Design, Patient Population	Results	Clinical Outcomes	Monocyte Function
**Randomized, unblinded, prospective study of sargramostim *vs* placebo: 40 patients with sepsis and a documented infection** (153)	• Sargramostim *vs* placebo: • More patients experienced cure/improvement of infection (14/18 *vs* 5/15; p=0.01) • More patients survived at 14 days (14/18 *vs* 10/15; p=0.10), 28 days (14/18 *vs* 9/15; p=0.53), and were discharged (12/18 *vs* 8/15; p=0.18) • Sargramostim increased mHLA-DR expression to a level that was not different from that of healthy controls (p=0.27) • Positive correlation between the amount of increase in HLA-DR expression and the clearance of infection (r=0.41; p=0.02)	• Sargramostim increased the proportion of patients whose infections were either cured or improved • Improvement in 28-day survival and hospital discharge in patients who received sargramostim (p=NS)	• Sargramostim normalized mHLA-DR expression • Increased mHLA-DR expression correlated with infection clearance
**Prospective, single-arm study of sargramostim: 4 children^a^ with recurrent infections** (154)	• Sargramostim treatment *vs* baseline: • Decreased number of repeated viral infection complaints (patients 1-3) • Increased diameter of induration for antigens tested *via* DHT (all patients) • Higher absolute monocyte counts at Week 12 (0.4–0.7 x 10^3^ *vs* 0.2–0.4 x 10^3^; p=NR)	• Sargramostim treatment reduced the number and severity of infections • Sargramostim improved immune function as reflected in positive DHT response	• Sargramostim treatment increased absolute monocyte counts at Week 12
**Randomized, double-blind, phase 2 study of sargramostim *vs* placebo: 38 patients with severe sepsis or septic shock and sepsis IP** (31)	• Sargramostim *vs* placebo: • Shorter ICU LOS (40.9 days *vs* 52.1 days; p=NS) • Shorter intrahospital LOS (58.8 days *vs* 68.9 days; p=NS) • Shorter time on ventilator (147.9 days *vs* 207.2 days; p=0.037) • Similar 28-day mortality (16% *vs* 21%; p=NS) • Higher Day 9 mHLA-DR (50,907 Ab/c *vs* 10,426 Ab/c; p<0.0001) • Higher proportion of patients with normalized mHLA-DR levels (100% *vs* 16%) • Higher TLR4-induced cytokine release at Day 9 (IL-6, p<0.05; IL-8, p<0.01)	• Time of mechanical ventilation, ICU LOS, and intrahospital LOS shorter in patients who received sargramostim • No significant change for 28-day mortality between the groups	• Sargramostim normalized mHLA-DR levels • Sargramostim treatment restored cytokine production
**Randomized placebo-controlled study of sargramostim *vs* placebo: 36 patients with severe sepsis and sepsis IP** (155)	• Sargramostim *vs* placebo: • Lower Day 9 kynurenine levels (p=0.009) • Similar 28-day mortality (17% *vs* 22%; p=0.9) • Lower Day 9 IDO activity (p=0.03) • Correlation with procalcitonin and IDO activity (r=0.56; p<0.0001) • Inverse correlation of mHLA-DR and IDO activity (r=-0.28; p=0.005)	• Sargramostim improved antibacterial defense as indicated by decreased IDO activity and reduction in kynurenine pathway catabolites	• Sargramostim treatment induced normalization of monocytic function that is accompanied with decreased levels of IDO activity and metabolites downstream of IDO
**Randomized, open label, phase 2 study of sargramostim *vs* SoC: 14 children with MODS and IP (defined by whole-blood *ex-vivo* LPS-stimulated TNF-α response)** (126)	• Sargramostim *vs* SoC: • No nosocomial infections in sargramostim-treated group (p<0.05) • For children who received SoC, IP reversal required > 7 days in the PICU • For children who received sargramostim: • IP reversal occurred in < 7 days • Rapid recovery of *ex-vivo* LPS-induced TNF-α production (200 pg/mL) compared with children who received SoC (p=0.001)	• Sargramostim reduced nosocomial infections without increasing systemic inflammation • Sargramostim-treated patients required fewer days in the PICU	• Sargramostim treatment restored monocyte responsiveness
**Randomized, double-blind, placebo-controlled study of sargramostim *vs* IFN-γ and placebo: 18 healthy volunteers, experimental endotoxemia leading to IP induced with *E. coli* endotoxin** (148)	• During the second LPS challenge: • Lower reduction in symptom scores with sargramostim (50%; p=0.03) *vs* placebo (72%; p=0.03) • Decrease in TNF-α release (from Visit 1 to Visit 2) with sargramostim (38% [-2 to 63, p=0.16]) and placebo (60% [48–71%; p= 0.03]) • During the treatment period, sargramostim *vs* placebo: • Stable mHLA-DR (85% to 94%; p=0.40 *vs* 80% to 76%; p=0.30) • Higher leukocyte counts (+21% *vs* -24%; p=0.009)	• Sargramostim treatment diminished a reduction in symptom score response to a second LPS exposure in experimental endotoxemia IP model	• Sargramostim treatment stabilized mHLA-DR and prevented a reduction in monocyte responsiveness after second LPS exposure
**Randomized, single-blinded, phase 2a study of sargramostim *vs* placebo: 38 patients with sepsis and impaired neutrophil function** (99)	• Sargramostim *vs* placebo: • Higher neutrophil function (phagocytosis ≥ 50%) at Day 6/7 (100% *vs* 44%; p=0.004) • Lower all-cause 30-day mortality (23.5% *vs* 28.6%; for those who received ≥ 2 doses of trial drug, 7.7% *vs* 30%) • Higher mHLA-DR at Day 2 (p<0.01)	• Sargramostim improved neutrophil function • Sargramostim improved all-cause 30-day mortality	• Sargramostim treatment was associated with a significant rise in mHLA-DR at Day 2 (p<0.01)

a Three children with severe and recurrent viral respiratory tract infections; 1 child with recurrent bacterial sepsis.

Ab/c, antibodies bound per cell; AUC, area under the curve; DHT, delayed hypersensitivity skin test; HLA-DR, human leukocyte antigen-DR isotype; ICU, intensive care unit; IDO, indoleamine 2,3-dioxygenase; IFN, interferon; IL, interleukin; IP, immunoparalysis; LOS, length of stay; LPS, lipopolysaccharide; mHLA-DR, monocyte human leukocyte antigen-DR; MODS, multiple organ dysfunction syndrome; NR, not reached; NS, not significant; PICU, pediatric intensive care unit; SoC, standard of care; TLR4, toll-like receptor 4; TNF, tumor necrosis factor.

## Discussion

While mHLA-DR, TNF-α secretion, and ALC each show promise as potentially useful biomarkers for sepsis IP, analytic validity of HLA-DR expression and its direct biologic linkage with MNP functional state make it attractive as a potential future gold standard for identification of sepsis IP (157, 158). Numerous publications, dating back 20 years, support use of either HLA-DR+ CD14+ cells or the actual number of HLA-DR proteins on CD14+ monocytes as clinically useful biomarkers for identifying patients with sepsis. Furthermore, sepsis IP severity might be detected by either low HLA-DR levels or diminishing HLA-DR levels during hospitalization. Nonetheless, while sepsis IP can be detected by diminished mHLA-DR expression, absence of either validated testing or an approved therapy to correct sepsis IP have thus far prevented widespread adoption of this biomarker.

Based on data presented here, we conclude therapeutic GM-CSF restores mHLA-DR levels and may improve clinical outcomes in patients with sepsis IP. Multiple trials of critically ill adults and children indicate that study treatment with sargramostim restored HLA-DR expression and immunocompetence. Furthermore, sargramostim led to trends toward improved clinical outcomes *via* reduced days of ICU stay and 28-day mortality. The GRACE-2 and SepTIC trials will further inform benefit from therapeutic GM-CSF (sargramostim) in sepsis IP.

## Author contributions

IJ, WPC, and EPR contributed equally to this work. All authors contributed to conceptualization and writing (drafting, reviewing, editing) of this manuscript. All authors contributed to the article and approved the submitted version.
